# Editorial: Cell therapy to tissue engineering: Cutting edge research in ocular surface regeneration

**DOI:** 10.3389/fmed.2023.1178946

**Published:** 2023-03-22

**Authors:** Sayan Basu

**Affiliations:** ^1^Shantilal Shanghvi Cornea Institute, LV Prasad Eye Institute, Hyderabad, Telangana, India; ^2^Brien Holden Eye Research Centre, LV Prasad Eye Institute, Hyderabad, Telangana, India

**Keywords:** stem cell, cornea, ocular surface, regenerative medicine, bioengineering, tissue engineering

The ocular surface is a unique sensory ecosystem consisting of the cornea, conjunctiva, and adnexal elements, which are coated by a thin layer of the pre-ocular tear film (1). There is a delicate physiological balance between the diverse epithelial sub-types that blend into each other at the muco-cutaneous junction and at the limbus (1, 2). A dynamic equilibrium is also maintained by the different types of glands, their secretions, the movement of the eyelids, and the lacrimal drainage apparatus. Any immunological or traumatic insult can disturb this fragile microenvironment and set into motion a domino effect of inflammation that ultimately leads to corneal damage and vision impairment. Unfortunately, corneal damage in ocular surface disease is usually not amenable to corneal transplantation. This peculiar limitation has spawned research into regenerative approaches including stem cells and bioengineering.

This special issue on the Research Topic of ocular surface regeneration includes some of the cutting edge developments in cell therapy and tissue engineering (Singh et al.; Ramos et al.; Chen et al.; Li et al.). Recent developments in regenerative medicine have opened the door to the use of novel therapeutics with encouraging outcomes. This includes ocular surface regeneration using limbal tissues, cultured cells, and biomaterials. The approach of stem cell-based therapy has been spectacularly successful in the management of blinding chronic sequelae of chemical injuries [Li et al.; (3)]. Once considered incurable, ocular surface disease due to limbal stem cell deficiency is perhaps one of the best examples of both successful bench to bedside translational research and surgical innovation in ophthalmology (4). Besides limbal epithelial stem cell transplantation, epithelial transdifferentiation approaches have also been used for treating ocular surface epitheliopathy using oral mucosal stem cells (5).

Now the horizon for application of cell therapy is being expanded to corneal opacification due to scarring using mesenchymal stem cells (6). This is the leading cause of corneal blindness in developing countries where the burden of corneal blindness is the greatest. Unlike in the West, most causes of blinding corneal opacification in these populations carry an extremely poor prognosis for corneal transplantation (7). Attempts are ongoing to develop hydrogels and 3-D printed corneas for the treatment of corneal stromal pathologies like trauma, infections, and keratoconus (8, 9). Proof-of-concept and early clinical application of endothelial cell therapy for corneal edema and endothelial dysfunction has already shown promise (10). It is very likely that in the near future the cornea will become the first tissue to have regenerative therapy for each individual element in the form of epithelial, stromal, and endothelial therapy.

There is also great interest in regenerative approaches for the lacrimal and meibomian glands, that are important adnexal elements and critical to the health of the ocular surface [Chen et al.; (11)]. Damage to the lacrimal gland is the main cause of aqueous tear deficiency, and currently only palliative medical therapy is available for the treatment of this chronic condition (12). Three major approaches are being adopted, in the form of repair, regeneration, or replacement of the lacrimal gland (11). These goals are quite audacious but if successful will bring relief to people suffering from dry eye disease. The regenerative approaches for the treatment of dry eye disease also include development of biomaterials, nanomedicine, hydrogels, and drug-eluting contact lenses [Singh et al.; (13)].

The ocular surface is exceptional in terms of the range of research that is being done for developing regenerative approaches for each individual element be it the cornea, the conjunctiva, or the lacrimal glands. These developments are exciting and could pave the way for disruptive new therapies in the future. Once of the challenges would be to achieve the right balance between efficacy, safety, and affordability. As researchers we must not lose sight of those who are most desperately in need of these new therapies. Therefore, an equal emphasis must be placed on developing low-cost products that are easy to deliver (14). Despite the technological sophistication, if the solution is not affordable or accessible, it will not be widely adapted or used.

## Author contributions

The author confirms being the sole contributor of this work and has approved it for publication.
